# Pituitary Metastasis From Urothelial Carcinoma: A Case Report and Review of the Diagnosis and Treatment of Pituitary Metastases

**DOI:** 10.7759/cureus.17574

**Published:** 2021-08-30

**Authors:** David Bailey, Christine Mau, Brad Zacharia

**Affiliations:** 1 Neurosurgery, Penn State Health Milton S. Hershey Medical Center, Hershey, USA

**Keywords:** pituitary metastasis, urothelial carcinoma, pituitary adenoma, brain metastasis, sellar metastasis

## Abstract

The sellar and parasellar regions are a rare site of brain metastasis, most commonly from breast and lung cancer. Pituitary metastasis (PM) often presents as the first sign of metastatic disease but may herald early disseminated cancer. The diagnosis of PM requires differentiation from a benign pituitary adenoma. Although this may be proven definitively via surgical biopsy, a constellation of clinical findings including oculomotor palsy, visual disturbances, retroorbital pain, and diabetes insipidus is more suggestive of PM. Imaging is neither sensitive nor specific for differentiation but may inform the broader clinical picture. Due to its rarity, treatment guidelines for PM lack consensus, often including a mixture of radiation and surgery. Gross resection is challenging because of the vascular, invasive nature of these lesions. Stereotactic radiosurgery may be used to good effect either alone or in addition to resection. Even with treatment, the prognosis is poor. In this article, we present the third reported case of urothelial carcinoma metastasis to the pituitary. In addition, we review the clinical presentation, diagnosis, and treatment options including surgical resection and radiosurgery.

## Introduction

The sellar and parasellar regions are relatively rare sites of brain metastasis, comprising between 0.14% and 28.1% of all brain metastasis [1]. These lesions are most commonly due to breast and lung cancer, though metastases have been reported from nearly every part of the body [1]. Pituitary metastases (PM) are often asymptomatic, presenting most commonly as a result of breast or lung cancer in the sixth or seventh decade of life [2].

When symptomatic, it is important to differentiate metastasis from benign lesions such as pituitary adenoma. The most common presenting symptoms of PM include headache, oculomotor dysfunction, vision changes, and diabetes insipidus (DI) [3]. The treatment of metastatic pituitary lesions involves a combination of surgical resection with adjuvant radiation therapy. The effectiveness of these treatment modalities has yet to be fully understood, though it seems to be limited to symptomatic relief [1,4-7]. Overall, the prognosis of PM is poor as this site of metastasis is often a herald of more widespread disease [5].

Here, we present a rare case of urothelial carcinoma metastasizing to the sellar region. This is an unusual pathology, with only two previously reported cases found on literature review [8,9]. In this report, we will review the presentation, natural history, and treatment of PM.

## Case presentation

**Figure 1:**
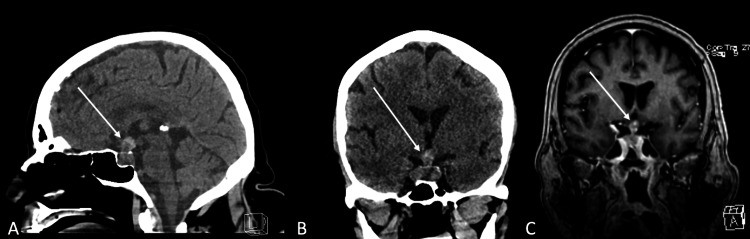
Presentation imaging. A, B: Noncontrast CT scan demonstrating a 2.4 × 1.2 cm lesion in the sella and suprasellar cistern. C: Postcontrast T1-weighted MRI demonstrating a lesion in the sella turcica with heterogeneous signal intensity and enhancement. CT: computed tomography; MRI: magnetic resonance imaging

A 61-year-old female with a medical history of high-grade urothelial carcinoma presented with altered mental status, poor oral intake, and falls for one week. She was first diagnosed with urothelial carcinoma five years prior, which was complicated by a number of relapses in situ, including a recent recurrence three months prior. She underwent transurethral resection and recently completed chemoradiation therapy in the preceding month. Upon presentation, a computed tomography (CT) scan revealed a 2.4 × 1.2 cm lesion in the sella and suprasellar cistern. Magnetic resonance imaging (MRI) demonstrated a lesion involving the sella turcica with heterogeneous signal intensity and enhancement (Figure 1). There was no indication of hemorrhage.

Laboratory results revealed low cortisol, thyroid-stimulating hormone (TSH), free T4, free T3, prolactin, luteinizing hormone (LH), and follicle-stimulating hormone (FSH). Diagnosis of anterior hypopituitarism was made in addition to central DI. The patient was taken for biopsy via a transsphenoidal transsellar approach. The lesion was firm and not able to be removed by suction, thus a complete resection was not attempted. Pathology revealed metastatic urothelial carcinoma which was positive for CK903, GATA3, and p63. The patient underwent CT of the chest, abdomen, and pelvis which did not reveal other metastatic sites. She then underwent stereotactic body radiation therapy (SBRT) consisting of 25 Gy in five fractions to the sellar region and was discharged.

The patient presented two months later with a left proximal diaphysis fracture. During this hospitalization, the patient developed acute adrenal insufficiency and severe epistaxis. A head CT revealed a large soft tissue mass in the sella extending to the sphenoid sinus. Further workup included CT chest, abdomen, and pelvis which revealed a significant burden of metastases within the liver, new from prior examinations.

**Figure 2:**
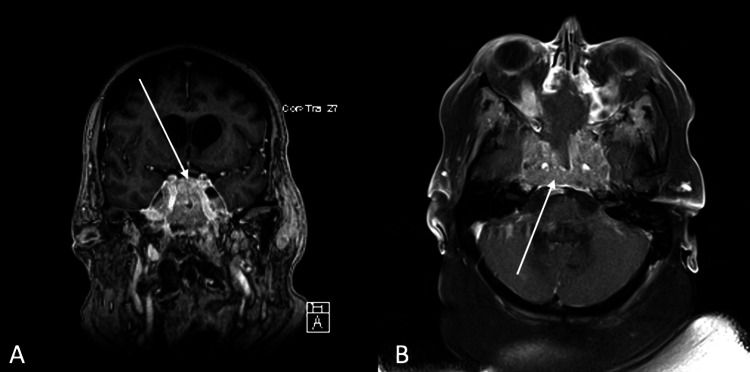
Two-month follow-up imaging. A, B: Postcontrast T1-weighted MRI demonstrating a 5.2 × 2.6 cm lesion. MRI: magnetic resonance imaging

A repeat MRI of the lesion revealed significant progression to 5.2 × 2.6 cm which now extended along the infundibulum of the pituitary gland to the floor of the third ventricle causing a mass effect on the optic chiasm (Figure 2). The mass demonstrated a diffuse, infiltrative, intermediate, T2-weighted signal. The soft tissue mass was associated with bony erosion in the central and anterior skull base. Furthermore, there was an invasion into the cavernous sinuses and encasement of the internal carotid artery bilaterally. Given the significant progression and diffuse metastatic disease, the family requested the patient be transitioned to hospice care before chemotherapy was initiated. The patient passed away shortly thereafter due to the rapid progression of the disease three months after her initial presentation.

## Discussion

Approximately 20-40% of cancer patients suffer from brain metastasis [10]. The incidence of intracranial metastasis seems to be increasing as a result of the increased sensitivity afforded by better imaging leading to earlier detection. In addition, improved treatment modalities have led to longer survival from primary cancers and an increase in the likelihood of metastasis [10]. While not commonly encountered in clinical practice, an autopsy series found that 3.6% of all cancer patients may have PM [11].

The most common metastatic tumors to the pituitary region are breast and lung, making up nearly two-thirds of all PM [1,3,4]. This high rate of breast cancer has been attributed to the hormone-rich environment, especially prolactin, present in the pituitary gland [4]. While these two etiologies are most common, PM has been reported from nearly every organ of the body [1,4].

Bladder metastasis to the brain is rare, accounting for approximately 0.6% of all brain metastasis [9]. Of these, only two previous metastasis to the pituitary region have been reported [8,9]. The latter case involved a 70-year-old male who presented with sudden-onset headaches, altered mental status, and visual disturbances four months following radical cystectomy for urothelial carcinoma. A CT scan revealed a suprasellar isointense mass with intratumor and intraventricular hemorrhages. A follow-up MRI demonstrated a well-circumscribed mass lesion in the intra- and suprasellar regions which was mildly enhancing with contrast. An endocrine workup demonstrated low FSH, LH, and TSH. The adrenocorticotropic hormone and prolactin levels were high while growth hormone was normal. The patient also had DI. A diagnosis of pituitary adenoma with hemorrhagic apoplexy was made. The patient underwent a transsphenoidal biopsy which revealed necrotic tissue and hematoma. They were unable to recover a tumor sample through the endoscopic approach; however, the patient reported improvement of visual disturbances. Therefore, further resection was pursued through a frontobasal interhemispheric and transtuberculum sellar approach. Surgical pathology revealed urothelial carcinoma. The patient refused further radiation therapy and died 3.5 months following resection and five months after presentation [9]. In the second case of urothelial cell carcinoma PM, a 41-year-old man with bladder cancer developed polyuria after his hypercalcemia was successfully managed. His PM of transitional cell carcinoma was documented on autopsy, which also showed widespread bone metastases [8].

Our case presented similar to the first case mentioned above, occurring shortly after the conclusion of treatment for urothelial carcinoma. Both abovementioned patients presented with symptoms of altered mental status, visual disturbances, and anterior and posterior hypopituitarism. In our case, we were able to obtain pathologic diagnosis through an endoscopic endonasal approach.

Between 6.8% and 16% of PM are symptomatic, likely leading to an underreporting of their incidence [11,12]. It is worth noting that a higher rate may be symptomatic, but any symptoms are overshadowed by the constitutional manifestations of the diffuse disease and are therefore not identified clinically [13].

In patients with known cancer, the discovery of a pituitary lesion should raise suspicion for a metastatic etiology. This is particularly important for surgical planning as many of these are not amenable to radical resection. When PM are discovered, they are often part of larger dissemination of the disease, often with five or more lesions and frequently involving the bone [5]. However, even if a search for additional metastasis is negative, it does not definitively rule out PM. Lesions to the pituitary gland may present as the first location of metastatic disease, which was seen in 44% of patients in a case series by Patel et al. [4]. Morita et al. also reported that 47% of those diagnosed with PM had only a pituitary lesion and no other discoverable site of metastasis. Notably, though, 76% of patients who presented with a solitary lesion later died from the disseminated disease [5]. This suggests that PM may herald wider dissemination of disease that may not be immediately evident. This was seen in the case presented here, where the initial presentation of the PM did not reveal further dissemination of the primary disease on a CT of the chest, abdomen, and pelvis. However, upon rehospitalization just two months later, the patient was discovered to have diffuse liver metastases.

The differentiation between benign and metastatic lesions may be predicted by analyzing clinical symptoms as well as imaging characteristics, though surgical biopsy is the only way to obtain a definitive diagnosis. While there is some debate over which PM symptoms are most common, the main manifestations include DI, oculomotor palsy, visual disturbances, and retro-orbital pain. The presence of all four of these symptoms has a sensitivity of 81.8% and a specificity of 93.5% for PM [3].

The presence of DI is specific for PM, only occurring in 1% of pituitary adenoma [1,3,5]. Comparatively, 84.6% of PM involve either the posterior or posterior and anterior pituitary gland together [14]. This predilection for the posterior pituitary is likely related to the direct connection between the posterior gland and systemic circulation [6]. While specific, the presence of DI is not sensitive and is not seen in up to 40% of PM [3]. More common etiologies of DI include craniopharyngioma, germ cell tumors, inflammatory pathologies, and idiopathic causes which must be ruled out in the absence of a known pituitary mass [15].

Imaging findings may also afford clues to whether a lesion is benign or metastatic, though there is significant overlap with the presentation of benign lesions making this modality nonspecific. A CT scan may show a hyperdense or isodense mass that is either homogeneously or heterogeneously enhancing, the latter if there is cystic degeneration, hemorrhage, or necrosis associated with the mass [1]. The MRI findings are nonspecific and may present as either iso- or hypointense on T1-weighted image and high intensity on T2-weighted image that is homogenously enhancing postgadolinium with the absence of high-signal intensity of the posterior lobe on T1-weighted image [1]. While these characteristics may be seen, they are not specific for PM versus pituitary adenoma [1]. Additionally, due to the rapid growth of PMs, the diaphragm may be spared. Imaging may demonstrate a dumbbell shape with a clear indentation at the level of the diaphragm sellae on sagittal imaging. While suggestive of a PM, the dumbbell sign is also commonly seen in a benign lesion [1]. Invasion of the cavernous sinus or infundibular recess by a suprasellar mass favors a PM because suprasellar adenomas typically push the infundibular recess posteriorly [16]. Also suggestive of PM is a linear enhancement of the infundibulum proximal to the tumor [16]. PM may demonstrate enlargement or enhancement of the pituitary stalk on CT or MRI, though this is only seen in 24% of cases [4,5].

There is a lack of consensus on formal treatment guidelines for PM. Most commonly, patients often undergo a combination of surgery and radiotherapy. Gross total resection is often challenging because PM are often heavily vascular, dense, invasive of the surrounding bone and the cavernous sinus, with frequent infiltration of the hypothalamus and optic nerves. The complexity of these lesions may lead to significant complications including new-onset pituitary insufficiency and oculomotor palsies [4]. Surgical resection is often performed through a transsphenoidal approach. This may be complicated by an intraoperative cerebrospinal fluid leak, though this risk has been decreased in recent years with the use of endoscopic techniques [6,17]. Less commonly, a cranial route may be used if the lesion is located at the upper part of the pituitary gland [6]. While surgical improvement of overall survival has yet to be clearly demonstrated, this treatment modality has been proven effective in improving the quality of life and relieving symptoms [1,5,6]. Decompression most commonly improves visual and oculomotor symptoms but may also relieve both posterior and anterior endocrine dysfunction [1].

In addition to surgery, radiation is a common treatment for PM with or without surgery [18]. A case series by Chon et al. demonstrated significant symptom relief with stereotactic radiosurgery. This series demonstrated improvement of DI and vision function when treated with radiosurgery without any new deficits [18]. In addition, case series by Kano et al. and Iwai et al. showed improvement of DI, cranial nerve dysfunction, and vision with radiosurgery [19,20]. However, in the follow-up analysis by Kano et al., 22% of patients developed new cranial nerve deficits and worsening anterior pituitary function, which was mainly attributed to tumor progression [19]. This calls into question the durability of this treatment modality despite the clear effectiveness in symptom amelioration [19].

While data are limited, there are several case series describing treatment for PM that are worth highlighting. Zacharia et al. found that extended endoscopic transsphenoidal approaches resulted in an 81% rate of gross total resection with overall survival of 16 months. They also demonstrated an 18-month progression-free survival when surgery was combined with radiotherapy, demonstrating excellent local control [17]. Burkhardt et al. in 2016 reported a gross total resection rate of 29%. Combined with adjuvant radiotherapy, patients had a mean overall survival of 16 months following the diagnosis of PM and 36 months after cancer diagnosis [7]. Patel et al. in 2020 reported the largest case series for the treatment of PM to date, consisting of 85 patients [4]. The authors reported significant symptom relief with both surgery and radiation. They also showed an improved overall survival rate in patients who received surgical treatment. The authors reported that while this may be related to the surgical treatment, it may be confounded by differences in patient characteristics, ultimately resulting in a susceptibility bias. They also suggested this result may be related to improved control of the systemic disease.

Overall, the prognosis for PM is poor as it signifies a more widely disseminated disease, which ultimately leads to mortality [5]. Regardless of the treatment strategy, PM has a poor median life expectancy. Recently, Patel et al. reported a median survival of 16.5 months, higher than previously reported, which they attributed to improved management of advanced cancer [4]. Positive prognostic factors include younger age at diagnosis, later metastasis to the pituitary, and smaller PM size [21].

## Conclusions

Presented here is an exceptionally rare case of PM from urothelial carcinoma which is only the third reported case from this primary etiology. The patient presented with a solitary metastatic lesion in the sella and suprasellar cistern. The lesion was not amenable to surgical resection and was treated with SBRT. The patient presented again two months later with significant liver metastasis and PM progression. The patient passed away soon thereafter due to rapid disease progression.

PM is most commonly a manifestation of breast or lung cancer, though instances have been reported from nearly all tissues. Diagnosis of PM requires differentiation from benign lesions. This may be done by reviewing the clinical picture and imaging findings, but tissue pathology is required for a confirmed diagnosis. Treatment of PM consists of a combination of radiation therapy and surgical resection which is indicated for palliation. Overall, the prognosis for PM is poor given the often widely disseminated nature of primary cancer. More reported cases would help clarify the appropriate treatment course for these rare but important cases.
